# Efectividad de las intervenciones para mitigar la influencia de las redes sociales en la anorexia y bulimia nerviosa: una revisión sistemática

**DOI:** 10.23938/ASSN.1074

**Published:** 2024-04-17

**Authors:** Noelia Lozano-Muñoz, Álvaro Borrallo-Riego, María Dolores Guerra-Martín

**Affiliations:** 1 Servicio Andaluz de Salud Centro de Salud de Constantina Sevilla España; 2 Universidad de Sevilla Facultad de Enfermería, Fisioterapia y Podología Departamento de Enfermería Sevilla España; 3 Instituto de Biomedicina de Sevilla (IBiS) Sevilla España

**Keywords:** Anorexia Nerviosa, Bulimia Nerviosa, Ensayo Clínico Controlado Aleatorizado, Redes Sociales, Revisión sistemática, Anorexia Nervosa, Bulimia Nervosa, Randomized Controlled Trial, Social Networking, Systematic review

## Abstract

**Fundamento:**

El objetivo es analizar el impacto de las intervenciones para mitigar la influencia de las redes sociales (RRSS) en la anorexia y bulimia nerviosas.

**Métodos:**

Revisión sistemática de las bases de datos PubMed, Scopus, PsycINFO y *Web of Science*. Se incluyeron ensayos clínicos aleatorizados, publicados entre 2013 y 2023, con puntuación mínima de 5 en la escala de calidad metodológica de Van Tulder.

**Resultados:**

Se seleccionaron ocho estudios desarrollados en centros de educación secundaria o universitaria -uno *online*- que incluían 5.084 participantes, principalmente mujeres jóvenes y adolescentes, con edad media entre 12 y 32 años. El impacto de las RRSS se estudió de forma global o centrándose en *Instagram, Facebook, Tik-Tok, Twitter* o *Snapchat* Se encontró una correlación positiva entre la exposición a ideales de belleza irreales en las RRSS y una mayor preocupación e insatisfacción por la imagen corporal. Todos los estudios utilizaron instrumentos para valorar la efectividad de las intervenciones. Las intervenciones permitieron reducir la influencia percibida por los medios y las RRSS, mejorar las valoraciones en la autopercepción y autoestima, disminuir los niveles de ansiedad y de internalización de ideal de belleza delgado, reducir las restricciones dietéticas y mejorar el uso de las RRSS.

**Conclusiones:**

Las intervenciones más duraderas lograron mejoras en satisfacción corporal (un año) y en síntomas depresivos (seis meses), especialmente en mujeres. Las intervenciones deben incluir atención a la autocrítica, autopercepción, autoestima, imagen corporal, manejo nutricional y alfabetización mediática.

## Abstract (en)

**Background:**

The study aim to analyze the impact of interventions to help mitigate the influence of social networks on anorexia and bulimia nervosa.

**Methodology:**

A systematic review was conducted using PubMed, Scopus, PsycINFO and Web of Science. The inclusion criteria were: randomized clinical trials, published between 2013 and 2023 with a score ≥ 5 points on the Van Tulder methodological quality scale.

**Results:**

Eight studies were selected, most carried out in secondary education centers or universities; one was online. The study sample consisted of 5,084 participants mainly young women and adolescents with an average age between 12 and 32 years. As for the social networks, some studies described their impact at a general level, while others focused on Instagram, Facebook, Tik-Tok, Twitter, and Snapchat. A positive correlation was found between the exposure to unrealistic beauty ideals found in social networks with greater concern and dissatisfaction with body image. All studies used instruments to assess the effectiveness of the interventions. The interventions helped reduce the influence of the media and social networks, improve self-perception and self-esteem assessments, reduce anxiety levels and internalization of the thin beauty ideal, reduce dietary restrictions, and make use of social networks differently.

**Conclusions:**

Longer-lasting interventions can improve body satisfaction (one year) and depressive symptoms (six months), especially in women. Interventions should include attention to self-criticism, self-perception, self-esteem, body image, nutritional management, and media literacy skills.

## INTRODUCCIÓN

En los últimos años se ha observado como la influencia de las redes sociales (RRSS) afectan al desarrollo de los trastornos de la conducta alimentaria y de la ingestión de alimentos (TCA)^1^. Los TCA se definen como una serie de psicopatologías caracterizadas por alteraciones en la ingesta, restricción de alimentos, episodios de atracones y excesiva preocupación por la figura corporal y/o por el peso^2^. En España, los casos de TCA se han incrementado un 30% respecto a niveles prepandemia de COVID-19^3^^,^^4^. Respecto a cifras mundiales, la OMS afirmaba en 2018 que la prevalencia de TCA ascendía a un 5%^5^, mientras que una revisión sistemática concluyó que la prevalencia de TCA se había duplicado entre los años 2000 y 2018, incrementándose del 3,4 al 7,8%^6^. Por todo ello, los TCA son considerados en la actualidad un problema de salud pública que suponen un grave riesgo para la salud. Si bien los TCA afectan en especial a la población adolescente, diversos autores describen que la población entre 40 y 60 años tiene un riesgo aumentado de sufrirlos^7^^,^^8^.

La anorexia nerviosa (AN) es un trastorno caracterizado por una pérdida de peso intencional, inducida y mantenida por el paciente^9^. El DSM-V establece dos subtipos de AN según el mecanismo de pérdida de peso: el restrictivo lo consigue a través de la dieta, el ayuno y/o la realización excesiva de ejercicio, mientras que en el tipo con atracón/purga existen vómitos autoprovocados de manera regular o un uso inadecuado de laxantes, diuréticos o enemas^2^. La bulimia nerviosa (BN) es un síndrome caracterizado por una preocupación importante por el control del peso corporal que se acompaña de episodios repetitivos de hiperingestión de alimentos seguida de vómitos y uso de purgantes^9^.

La prevalencia muestra amplias diferencias en función del grupo de edad y del sexo, siendo muy superior en mujeres jóvenes (AN: 0,1-2%; BN: 0,37-2,98%). En Europa, la prevalencia estimada de mujeres que padecen anorexia nerviosa es de un 1-4%, mientras que de bulimia nerviosa es del 1-2%^10^. A nivel mundial la prevalencia de AN se sitúa en un 0,3%-1% y de BN del 1-3%^11^.

Cada vez más personas de todas las edades hacen uso de las RRSS^12^^,^^13^. Según el informe Digital 2024, la base global de usuarios se incrementó un 5,6% y se alcanzaron los cinco billones de usuarios activos en las RRSS, lo que representa el 62,3% de la población mundial^14^. *Facebook* continúa siendo la red social más utilizada a nivel mundial, y durante 2023 incorporó 91 millones de nuevos usuarios^14^, a pesar del crecimiento exponencial de nuevas RRSS como *Instagram* y *Tik-Tok*, que ocupan el segundo y tercer puesto de RRSS con más usuarios^15^. A pesar de que *Tik-Tok* ostenta el tiempo promedio de uso por usuarios más alto de todas las RRSS, *Instagram* se afianza como la RRSS favorita a nivel mundial, respaldada por un 16,5% de los internautas entre los 16 y 64 años^14^. Otra plataforma en auge es YouTube, servicio gratuito de almacenamiento, administración y difusión de videos, que en 2023 recibió 33,04 billones de visitas, ocupando el segundo puesto de sitios web más visitados a nivel mundial, superado tan solo por Google^14^^,^^16^. *Twitter* (ahora X) también se ha convertido en una de las RRSS más utilizadas en la actualidad, donde millones de usuarios pueden compartir intereses, contenidos e intercambiar experiencias y opiniones^17^.

El uso de las RRSS presenta aspectos positivos, como ofrecer nuevas oportunidades de socialización, facilitar la búsqueda y encuentro de empleo, y brindar un mayor acceso a información positiva en materia de salud^1^, conformando una estructura que fomenta la interacción social, incluidos los vínculos de reciprocidad entre personas^18^. También se han descrito aspectos negativos derivados de su uso, como su contribución a la auto asignación de modelos de cuerpos ideales y a la publicidad hipersexualizada^19^. Diversos estudios han encontrado una asociación significativa entre el uso de RRSS y niveles más elevados de ejercicio estricto, de baja autoestima y de insatisfacción corporal, lo que puede aumentar el riesgo de TCA^20^^,^^21^. Debe tenerse en cuenta que, últimamente, en las RRSS se tiende a reemplazar la escritura por contenido visual como fotografías y vídeos divulgativos^15^^,^^22^.

En el contexto de las RRSS merece atención el rol de los *influencers*, que en los últimos años se han convertido en importantes fuentes de información y en fenómenos publicitarios por su impacto, influencia e inspiración en la audiencia que les sigue, especialmente entre los más jóvenes^23^^-^^25^. Los jóvenes de la muestra del estudio de Beatriz Feijoo y col^24^ reconocieron que las principales colaboraciones que realizan los *influencers* con marcas comerciales están relacionadas con el sector del cuidado y la alimentación. La autopercepción y la percepción corporal puede verse afectada por los contenidos visualizados en las distintas RRSS, lo que incentiva una cultura inspirada en la imagen y en la idealización de determinados cánones físicos^23^.

Basándonos en todo lo descrito, la presente revisión sistemática se plantea analizar la efectividad de las intervenciones para mitigar la influencia de las RRSS en la anorexia y bulimia nerviosas.

## MATERIAL Y MÉTODOS

*Diseño y Registro*. Siguiendo las recomendaciones del Manual Cochrane^26^ y las normas PRISMA^27^, se llevó a cabo una revisión sistemática, por pares, que se registró en PROSPERO con el identificador CRD42023385306.

*Criterios de elegibilidad*. Los criterios de inclusión fueron: ensayos clínicos aleatorizados (ECA) publicados en los últimos diez años (2013-2023), que tratasen la relación entre AN/BN y el uso de las RRSS e incluyeran intervenciones para analizar su efectividad. Fueron excluidos los resúmenes de congresos, estudios en animales o in vitro y los estudios que no aportaron resultados.

*Estrategia de búsqueda*. La búsqueda se llevó a cabo entre febrero y abril de 2023 en las bases de datos PubMed, SCOPUS, PsycINFO y Web of Science. Se utilizaron palabras claves y sinónimos para maximizar la sensibilidad de la búsqueda^28^, con la siguiente estrategia: *(“Social Networking” OR “Online Social Networking”) AND (“Anorexia” OR “Anorexia Nervosa” OR “Bulimia” OR “Bulimia Nervosa”)*.

*Selección de estudios.* Los estudios se recuperaron a través de las cuatro bases de datos y, una vez descartados los duplicados, dos revisores realizaron tres cribados de forma independiente. En el primer cribado se tuvo en cuenta el título y resumen, y en el segundo se procedió a la lectura a texto completo; en el tercero se evaluó la calidad metodológica de los estudios.

*Calidad de los estudios*. Dos revisores independientes evaluaron la calidad de los estudios mediante la escala Van Tulder, conformada por once ítems: A. ¿Fue adecuado el método de aleatorización?, B. ¿Se ocultó la asignación al tratamiento?, C. ¿Eran los grupos similares al inicio con respecto a los indicadores pronósticos más importantes?, D. ¿Los pacientes estaban cegados a la intervención?, E. ¿El provedor de atención estaba cegado a la intervención?, F. ¿El evaluador de resultados estaba cegado a la intervención?, G. ¿Se evitaron las co-intervenciones o fueron similares?, H. ¿Fue aceptable el cumplimiento en todos los grupos?, I. ¿Se describió y fue aceptable la tasa de abandono?, J. ¿Fue similar el momento de la evaluación de resultados en todos los grupos?, K. ¿En análisis incluyó un análisis por intención de tratar? Cada ítem presenta tres opciones de respuesta (sí, no, no lo sé), sumando un punto por cada respuesta afirmativa. Una puntuación igual o superior a cinco se considera calidad alta^29^^,^^30^.

*Extracción y síntesis de datos*. Dos autores extrajeron de forma independiente los datos relevantes de los estudios seleccionados para su inclusión elaborando una Tabla específica para ello siguiendo las recomendaciones de Rafael del Pino Casado y col^31^. Los datos extraídos fueron cotejados y discutidos para llegar a un acuerdo entre ambos revisores. De cada uno de los estudios seleccionados se recopilaron los siguientes datos: 1) autores y año de publicación, 2) objetivo, 3) datos de la intervención, 4) muestra, 5) instrumentos de medida. Además, se recogieron los datos relacionados con la efectividad de las intervenciones realizadas según su pertenencia al grupo intervención o al grupo control.

## RESULTADOS

### Características de los estudios

**Figura 1:**
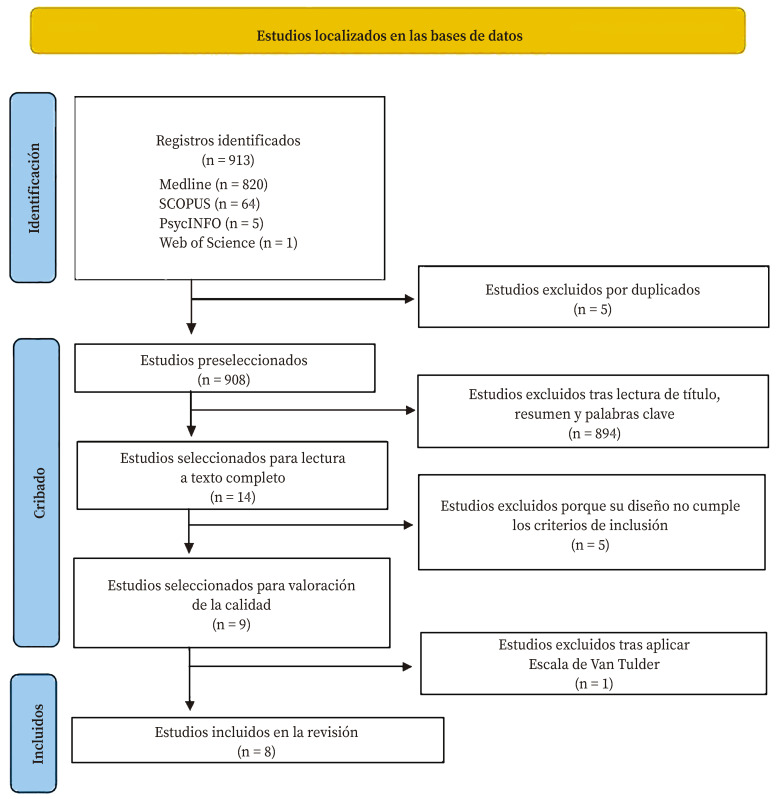
Diagrama de flujo del proceso de selección de estudios.

Las estrategias iniciales de búsqueda identificaron un total de 913 estudios. Tras el primer cribado se seleccionaron 14 y tras el segundo nueve; en el tercero, tras aplicar la escala de Van Tulder, se seleccionaron ocho estudios^32^^-^^39^ (Figura 1, Tabla 1).

Respecto a la calidad de los nueve estudios seleccionados tras los dos cribados iniciales, uno de ellos fue excluido de la revisión por obtener una puntuación inferior a 5 puntos en la escala de Val Tulder. Ninguno de los ocho estudios incluidos en la revisión cumplió los criterios B, E y F de la escala (Tabla 1).

**Tabla 1:** Calidad de los estudios cribados según la escala de Van Tulder

Autor/es y Año	Criterios de la escala	Puntuación
		Total
Bell y col, 2022	ACGHIJ	6
De Valle y col, 2022	ACGHIK	6
Kinkel-Ram y col, 2022	ACGHI	5
Tang y col, 2022	ACGHI	5
Gordon y col, 2021	ACGHIJK	7
Svantorp-Tveiten y col, 2021	ACGHIK	6
McLean y col, 2019	CHIJK	5
Mabe y col, 2014	CGHIJ	5

En la Tabla 2 se muestran las características de los ocho estudios seleccionados: 1. Autoría, año de publicación y país. 2. Muestra: tamaño, sexo y edad media. 3. Intervención, sesiones, duración y lugar. 4. Objetivo, instrumentos de medida, tiempos de aplicación y resultados. Tres de los estudios se llevaron a cabo en Oceanía^33^^,^^36^^,^^38^, otros tres en América del Norte^34^^,^^35^^,^^39^ y dos en Europa^32^^,^^37^. El tamaño de muestra de seis estudios osciló entre 100 y 500 participantes^32^^-^^36^^,^^38^ y en dos fue superior a 500^37^^,^^39^. La muestra total estuvo compuesta por 5.084 participantes, 72,85% mujeres.

**Tabla 2:** Características de los estudios incluidos en la revisión

Autoría	Muestra	Intervención	Objetivo
Año	% mujeres	Sesiones y duración	Instrumentos de medida
País	Edad media	Lugar	Tiempos de aplicación
			Resultados
Bell y col	- n= 268	- GI: Intervención *Digital Bodies* para mejorar la imagen corporal de los adolescentes y desafiar los ideales de apariencia poco realistas, tal y como aparecen en las RRSS.	- Evaluar la eficacia de *Digital Bodies*
202232	GI: n=134	- GC: sin intervención	- BSS, subescala *The Thinness and Athletic internalization* del SATAQ-4, SOQ
Inglaterra	GC: n=134	- Una sesión de 1h.	- T1: al inicio; T2: a la semana; T3: tras ocho semanas
	- 53%	- Escuela secundaria (mixta).	- Las mujeres del GI mostraron respecto al GC:
	- 12,80 años		• más internalización del ideal de apariencia delgada, mayor auto objetivación, y menor internalización del ideal atlético y satisfacción corporal que los hombres.
			• en T2, reducción de internalización del ideal de apariencia delgada. Sin efecto en hombres.
			• mayor satisfacción con imagen corporal, mantenida en T3.
De Valle y col	- n=130	- Dos módulos de intervención:	- Evaluar viabilidad, aceptabilidad y eficacia de una intervención autocrítica que aborde el vínculo entre el uso de las RRSS motivado por la apariencia y el riesgo de trastornos alimentarios.
202233	GIac: n=44	• GI autocrítica (GIa): 1.Psicoeducación sobre autocrítica y autocompasión; 2. Autocompasión e identificación de barreras; 3. Relevancia personal de generar autocompasión; 4.RRSS: uso para resolver problemas y reducción de su impacto en la imagen corporal.	- MSMU-5-item *appearance subscale*, PACS-R, REDEQ - 9-item *self-criticism subscale*, BI-AAQ, EDE-Q-versión corta.
Australia	GIc: n=43	• GI curación de RRSS (GIc): 1.Psicoeducación en RRSS y salud mental; 2.Discutir la idealización del contenido de las RRSS, y preguntar sobre ese contenido para mejorar la alfabetización; 3.Identificar lo que gusta/disgusta de las RRSS; 4.Reflexión sobre ello.	- T1: a la semana de la aleatorización; T2: a las dos semanas de la aleatorización.
	GC: n=43	- GC: lista de espera.	- Sin diferencias significativas entre GIac y GIc:
	- 89,2%	- Ocho sesiones de 15 minutos por submódulo.	• el GIa disminuyó la motivación de uso de RRSS basada en la apariencia y aumentó la flexibilidad respecto a su imagen corporal frente al GC
	- 19,29 años	- Universidad	• el GIc disminuyó la comparación de apariencia en RRSS respecto del GC.
			• resultados mantenidos en T2.
Kinkel-Ram y col	- n=434	- GI: visionado de 4 vídeos de 2 a 3 minutos de páginas de *Instagram* con imágenes de alimentos de baja densidad calórica.	- Evaluar cómo la visualización de imágenes de alimentos bajos en calorías afecta a la satisfacción corporal, la autoestima y si es propicio a la introducción en una alimentación desordenada.
202234	- 100%	- GC: visionado de imágenes relacionadas con viajes.	- SSES, EDBI, BISS.
EEUU	- Medio Oeste:	- 8-12 minutos de duración.	- T1: previo a la intervención; T2: tras la intervención.
	n=220	- *Online*.	- Sin diferencias entre GI y GC:
	18,59 años		• El GI del Medio Oeste presentó actitudes más cercanas a trastornos alimentarios frente al GC, que la del Medio Sureste.
	- Medio sureste:		• Raza, etnia y orientación sexual no influyeron en satisfacción de la imagen corporal ni autoestima.
	n=214		
	19,36 años		
	- no especifica n de GI y GC		
Tang y col 2022^35^	- n=132	- GI: visionado de imágenes de alimentos de bajo aporte calórico y relacionadas con el cuerpo en *Instagram*, *Facebook*, *Tik-Tok*, *Twitter* y *Snapchat*	- Conocer el efecto de RRSS sobre insatisfacción corporal, actitudes y comportamientos de alimentación y actividad física de las madres posparto.
Canadá	GI: n=65	- GC: visionado de consejos infantiles sobre una buena alimentación	- EDI - 9-items *body satisfaction subscale*, BISS, EAT-26, IPAQ-SF, DEBQ.
	GC: n=67	- 15 publicaciones diarias durante 5 días consecutivos.	- T1: al inicio: T2: al mes.
	- 100% 0-6 meses posparto	- *Online*.	- El GI presentó:
	- 32,5 años		• mayor insatisfacción corporal y peor imagen corporal
			• mayor inspiración a ser físicamente activas (en T1)
			• menor inspiración hacia una alimentación saludable
			• mejor alimentación y conductas alimentarias menos restringidas
Gordon y col 2021^36^	- n=489	- GI: *SoMe*, programa de imagen corporal, dieta y bienestar de alfabetización en RRSS para adolescentes.	- Probar eficacia de *SoMe* para mejorar resultados de imagen corporal y bienestar en adolescentes.
Australia	GI: 259	- GC: sin intervención.	- EDE-Q, DEBQ, BCI, CESDR-10, RSES, SATAQ-3, SATAQ-4, UPACS.
	GC: 230	- Cuatro sesiones de 50 minutos cada una.	- T1: prueba inicial; T2: a la semana; T3: a los seis meses; T4: a los 12 meses.
	- 53,38%	- Escuela secundaria.	- El GI obtuvo:
	- 12,77 años		• mayor satisfacción corporal (mantenida en T4)
			• mejor evolución de los síntomas depresivos en mujeres (mantenida en T3)
			• mejores resultados respecto a restricción dietética (en T2).
			• menor impulso de aumentar musculatura e interiorización del ideal de apariencia delgada (en T2)
			• menor autoestima
Svantorp-Tveiten y col 2021^37^	- n=2.446	- GI (programa HBI): fomentar la autocrítica para reducir los trastornos alimentarios y aumentar la imagen corporal. Tres sesiones: 1.Imagen corporal. 2.Uso de las RRSS y 3.Estilos de vida saludable. Evaluó también ansiedad, flexibilidad mental y preocupación por la imagen corporal.	- Investigar efecto de intervención escolar para reducir el riesgo y mejorar los factores protectores respecto al desarrollo de trastornos alimentarios en niños y niñas y para promover la comprensión crítica de la influencia de los medios sociales.
Noruega	GI: 1.499	- GC: sin intervención.	- EDE-Q, RSES, SCL-10, BI-AAQ, SATAQ-4, DLS.
	GC: 947	- Tres sesiones de 90 minutos cada una.	- T1: en el momento de la aplicación; T2: a los tres meses; T3: a los doce meses.
	- 58% mujeres	- Escuela secundaria (públicas y privadas).	- El GI presentó:
	- 17 años		• menor tendencia hacia los trastornos alimentarios (solo en mujeres en T2 y T3)
			• mayor autoestima
			• menor angustia mental
			• menor interiorización del ideal de delgadez extremo
			• mayor flexibilidad sobre la imagen corporal (en hombres hasta T2, en mujeres en T3).
McLean y col 2019^38^	- n=255	Intervención *Happy Being Me*:	- Examinar cambios en la alfabetización mediática y los programas de comparación de apariencia derivados de *Happy Being Me*.
Australia	GImd: 99	- GI HBM-*media*: 1.Técnicas de manipulación de los medios para divulgar el cuerpo delgado como ideal de belleza. 2.Empoderar mediante autocrítica y reconocimiento las cualidades positivas. 3.Desarrollar las técnicas subyacentes y manipuladoras de los medios; y exponer presentaciones para promover la salud.	- EDI, DEBQ, SATAQ-3, PACS, UPCAS, DACS, MAQ -*realism skepticism* (2 items), MAQ *critical thinking about media messages* (6 items), FNAES.
	GIcmp: 81	- GI HBM-*comparison*: 1.Conceptos y tendencia hacia la comparación con los iguales. 2.Resultados negativos de la comparación entre personas. 3.Presentaciones sobre cómo actuar ante situaciones de comparación.	- T1: a la aplicación; T2: tras la intervención; T3: a los tres meses.
	GCea: 75	- GC HBM-*eating*: promoción de conductas alimentarias saludables, sin dietas y comiendo ante sensación de hambre.	- El GI HBM-*media* presentó:
	- 100%	- Tres sesiones. Sin especificar duración.	• mayor restricción dietética respecto al GCea. aumento del pensamiento crítico
	- 13,09 años	- Escuela secundaria.	• cambios en la cultura del ideal de belleza delgado
			• mejoría en los síntomas bulímicos
			• aumento en autoestima.
Mabe y col 2014^39^	- Estudio 1 n=960	En el Estudio 1 se preguntó sobre uso de *Facebook* (de 0 a >7h), y se pasó la escala EAT-26 en dos semestres distintos	- Relación del uso de *Facebook* con patología alimentaria (estudio 1) y con cambios temporales en factores de riesgo de trastornos alimentarios (estudio 2).
EEUU	100%	Las jóvenes con uso al menos semanal se reclutaron para el Estudio 2:	- Estudio 1: EAT-26; estudio 2: EAT-26, STAI, VAS.
	18,44 años	- GI: en su cuenta de *Facebook*.	- T1: antes de la sesión; T2: tras la sesión.
	- Estudio 2 n=84	- GC: Wikipedia y YouTube investigando sobre el ocelote (animal de la selva tropical).	- El GI mostró mayor:
	100%	- Una sesión de 20 minutos.	• tendencia a la comparación entre iguales, mayor ansiedad (mientras que el GC logró una disminución significativa de la ansiedad)
	18,39 años	- Universidad.	• preocupación por imagen corporal/peso
	- no especifica n de GI y GC		• tendencia a trastornos alimentarios
			• importancia dada respecto a recibir comentarios y *likes* sobre su estado y fotos

GI: grupo intervención; GC: grupo control; h: horas. BCI: *Body Change Inventory*; BI-AAQ: *Body Imagen Acceptance and Action Questionnaire*; BISS: *Body Imagen States Scale*; BSS: *Body Satisfaction Scale*; CESDR-10: *Centre for Epidemiological Studies Depression Scale Revised*; DACS: *Downward Physical Appearance Comparison Scale*; DEBQ: *Dutch Eating Behaviour Questionnaire*; DLS: *Dynamic Light Scattering*; EAT-26: *Eating Attitudes Test*; EDBI: *Eating Disorder Behavior Intentions Scale*; EDE-Q: *Eating Disorder Examination Questionnaire;* EDI*: Eating Disorder Inventory*; FNAES: *Fear of Negative Appearance Evaluation Scale*; IPAQ-SF: *International Physical Activity Questionnaire - Short Form*; MAQ: *Media Attitudes Questionnaire*; MSMU: *Motivations for Social Media Use Scale*; PACS-R: *Physical Appearance Comparison Scale-Revised*; REDEQ: *Reconstructed Depressive Experiences Questionnaire*; RSES: *Rosenberg Self-Esteem Scale*; SATAQ-3: *The Sociocultural Attitudes Towards Appearance Scale-3*; SATAQ-4: *Sociocultural Attitudes Towards Appearance Questionnaire*; SCL-10: Hopkins *Symptom Checklist*; SOQ: *Self-Objectification Questionnaire*; SSES: *State Self-Esteem Scale*; STAI: *State Trait Anxiety Inventory*; UPCAS: *Upward Physical Appearance Comparison Scale*; VAS: *Visual Analog Scales.*

Cuatro estudios estaban conformados exclusivamente por mujeres^34^^,^^35^^,^^38^^,^^39^, y el resto por participantes de ambos sexos^32^^,^^33^^,^^36^^,^^37^. La muestra estuvo constituida mayoritariamente por adolescentes y adultos jóvenes, siendo la edad media más baja descrita de 12,77 años^36^ y la más alta de 32,5 años^35^. En tres de los estudios los participantes tenían una edad media entre los 12-15 años^32^^,^^36^^,^^38^ y en cuatro osciló entre los 16-20 años^33^^,^^34^^,^^37^^,^^39^; solo en uno de los estudios fue superior a 30 años^35^.

Cinco estudios describieron el impacto de las RRSS a nivel general, sin centrar su investigación en una red en particular^32^^,^^33^^,^^36^^-^^38^, mientras que tres de los estudios se centraron en alguna RRSS: Shruti S Kinkel-Ram y col^34^ en *Instagram*, Annalise G Mabe y col^39^ en *Facebook*, y Lisa Tang y col^35^ en *Instagram*, *Facebook, Tik-Tok, Twitter* y *Snapchat*.

Los estudios aplicaron tres^32^^,^^34^, cuatro^39^, cinco^33^^,^^35^, seis^37^ u ocho^36^^,^^38^ escalas para estudiar distintos aspectos:

Para conocer la satisfacción, flexibilidad y/o estado de la imagen corporal de los participantes, cinco estudios utilizaron las siguientes escalas: *Body Satisfaction Scale* (BSS)^32^, *Body Image Acceptance and Action Questionnaire* (BI-AAQ)^33^^,^^37^, *Body Image States Scale* (BISS)^34^^,^^35^, *Body Change Inventory* (BCI)^36^, y la subescala de satisfacción corporal de la *Eating Disorder Inventory* (EDI)^35^.
Otros cinco estudios evaluaron los aspectos relacionados con la apariencia mediante *Sociocultural Attitudes Towards Appearance Questionnaire* (SATAQ-4)^32^^,^^36^^,^^37^, *Physical Appearance Comparison Scale-Revised* (PACS-R)^33^^,^^38^, *Sociocultural Attitudes Towards Appearance Scale-3* (SATAQ-3)^36^^,^^38^, *Upward Physical Appearance Comparison Scale* (UPACS)^36^^,^^38^, *Fear of Negative Appearance Evaluation Scale* (FNAES)^38^, *Downward Physical Appearance Comparison Scale* (DACS)^38^, y la subescala centrada en la apariencia de la *Motivations for Social Media Use Scale* (MSMU)^33^.
Seis estudios evaluaron el riesgo de sufrir trastorno alimentario y/o el comportamiento alimentario: *Eating Disorder Examination Questionnaire* (EDE-Q)^33^^,^^36^^,^^37^, *Eating Disorder Behaviour Intentions Scale* (EDBI)^34^, *Eating Attitudes Test* (EAT-26)^35^^,^^39^, *Dutch Eating Behaviour Questionnaire* (DEBQ)^35^^,^^36^^,^^38^ y la *Eating Disorders Inventory* (EDI)^38^.
Se utilizaron otras escalas para medir autoestima: *Self-Objectification Questionnaire* (SOQ)^32^ y la *State Self-Esteem Scale* (SSES)^34^, nivel de autocrítica: subescala de la *Reconstructed Depressive Experiences Questionnaire* (REDEQ)^33^, ansiedad: *Rosenberg Self-Esteem Scale* (RSES)^36^^,^^37^ y *Sate Trait Anxiety Inventory* (STAI)^39^ 4), depresión: *Centre for Epidemiological Studies Depressión Scale Revised* (CESDR-10)^36^, actividad física: *International Physical Activity Questionnaire-Short Form* (IPAQ-SF)^35^, y la actitud ante los medios de comunicación: *Media Attitudes Questionnaire* (MAQ)^38^.

Cinco estudios observaron que el nivel de exposición a ideales de belleza irreales en RRSS mostraba una correlación positiva con un mayor nivel de preocupación por la imagen corporal, una menor satisfacción corporal y la internalización del ideal de apariencia delgada, aspectos que -según estos estudios- se asociarían a un mayor riesgo de sufrir TCA^33^^-^^36^^,^^38^. La interiorización del ideal de belleza delgado y la auto objetivación son mayores en mujeres^32^^,^^33^.

Dos de los estudios observaron que los participantes de dan mucha importancia a los comentarios y “*me gusta*” recibidos por sus publicaciones en las RRSS, aumentando la tendencia a la comparación entre iguales y, en caso de no recibir la respuesta deseada a las publicaciones, mostrando niveles elevados de ansiedad o reducidos de autoestima^37^^,^^39^.

### Descripción de las intervenciones

Todos los estudios se desarrollaron en centros de educación secundaria o centros universitarios, a excepción de uno desarrollado íntegramente *online*^35^.

Mientras siete de los estudios llevaron a cabo intervenciones colectivas y dirigidas a poblaciones específicas (como estudiantes o embarazadas)^32^^-^^34^^,^^36^^-^^39^, tan solo uno la aplicó individualmente tras captar participantes a través de plataformas *online*^35^.

Las intervenciones aplicadas incluían, en todos los casos, aspectos relacionados con la importancia de informar, formar y/o educar a la población para aumentar los conocimientos sobre los trastornos alimentarios. Cinco estudios pusieron en marcha programas de autocrítica y alfabetización mediática^32^^,^^33^^,^^36^^-^^38^, tres utilizaron programas de visualización de elementos relacionados con el cuerpo, la delgadez y la pérdida de peso^34^^,^^35^^,^^39^, y uno abordó aspectos centrados en la imagen corporal, manejo nutricional y autoestima^36^.

En todos los estudios se incluyeron intervenciones de carácter educativo para mejorar el nivel de salud, aplicándose talleres interactivos^32^^,^^33^^,^^37^, cuestionarios e imágenes de impacto de forma *online*^34^^,^^35^^,^^39^ o charlas sobre prevención y promoción de la salud^32^. Los estudios que desarrollaron talleres interactivos^32^^,^^33^^,^^37^ pasaron en primer lugar un cuestionario para conocer las preocupaciones e ideales de los participantes, y seguidamente llevaron a cabo los talleres donde expusieron los contenidos relacionados con la imagen corporal, la influencia de sus iguales o las estrategias que podían utilizarse para aumentar la autoestima. Además, dos de estos estudios^32^^,^^37^ presentaron propuestas de los propios participantes a fin de implementar cambios en su vida en relación con la autopercepción y la tendencia a la comparación. En tres estudios^34^^,^^35^^,^^39^, y tras cumplimentar una encuesta expresando sus preocupaciones y completar las escalas definidas según la metodología de cada estudio (Tabla 2), los participantes visualizaron diferentes fotografías y vídeos incorporadas en publicaciones de RRSS según el grupo de asignación (intervención o control) para, finalmente, cumplimentar de nuevo los cuestionarios y escalas correspondientes para comparar los resultados con los obtenidos al inicio.

Las evaluaciones se realizaron al inicio de la intervención en todos los estudios, y se volvieron a realizar tras la intervención^34^^,^^39^, a las dos semanas^33^, al mes^35^, a los dos meses^32^, a los tres meses^38^ e incluso al año^36^^,^^37^.

### Efectividad de las intervenciones

Cinco estudios describieron una correlación positiva entre la exposición a ideales de belleza irreales en RRSS y una mayor preocupación por la imagen corporal, la internalización del ideal de apariencia delgada, y una disminución en la satisfacción corporal, aspectos que, según estos estudios, generan un mayor riesgo de sufrir trastornos alimentarios^32^^-^^36^^,^^38^. Las intervenciones realizadas en cinco estudios fueron efectivas para mejorar la satisfacción con la imagen corporal y la autoestima de los participantes, reducir la internalización de apariencia delgada, disminuir los síntomas depresivos y la angustia mental, y mejorar los resultados en cuanto a la restricción dietética^32^^,^^33^^,^^36^^,^^37^^,^^38^.

Las intervenciones focalizadas en la autocrítica de los participantes^32^^,^^33^^,^^36^^-^^38^ fueron efectivas para aumentar la capacidad de razonamiento autocrítico, lo que logró que los participantes se sintieran menos influidos por los medios y las RRSS. Además, permitieron disminuir la comparación entre iguales en las RRSS^33^, e incentivar una mejor valoración de su autopercepción y autoestima^32^^,^^37^. Estos dos últimos estudios^32^^,^^37^ detectaron diferencias significativas entre las variables autopercepción y autoestima según el sexo, observando que las intervenciones beneficiaron más a las mujeres que a los hombres^32^^,^^37^.

Mientras que el efecto de la mayoría de intervenciones no se midió o duró más de una semana, el efecto sobre la satisfacción con la imagen corporal se mantuvo incluso un año^36^^,^^37^ y la disminución de síntomas depresivos seis meses, especialmente en mujeres.

## DISCUSIÓN

A través de la presente revisión se ha analizado la efectividad de las intervenciones para mitigar la influencia de las RRSS en la AN y BN.

El tamaño muestral del 75% de los estudios seleccionados osciló entre 100 y 500 participantes, similar al referenciado por Jorge Emiro Restrepo y Tatiana Castañeda Quirama^40^, que analizaron la relación entre el riesgo de TCA y el uso de las RRSS en 337 mujeres, y por Lidia María Ortiz y col^41^, que estudiaron la prevalencia de los TCA -incluyendo aspectos relacionados con las RRSS- en 469 adolescentes.

La mayoría de los participantes fueron mujeres (72,9%), en consonancia con otros estudios sobre la misma temática, con porcentajes entre el 54%^41^ y el 83,9%^42^.

La composición mayoritaria de la muestra por adolescentes (97,99%) coincide con otros estudios centrados en el análisis de la influencia de las RRSS en la población en edad escolar y en la adolescencia^40^^,^^41^^,^^43^^,^^44^. No obstante, en la actualidad se está investigando con población de mayor edad^7^^,^^8^.

Al igual que otras investigaciones centradas en el análisis de la asociación entre las RRSS y los TCA^41^^,^^43^^,^^45^, todos los estudios de la revisión excepto uno^35^ se desarrollaron en centros de educación secundaria o universitaria. Y aunque los estudios incluidos proceden de Oceanía^33^^,^^36^^,^^38^, América del Norte^34^^,^^35^^,^^39^ y Europa^32^^,^^37^

, en la literatura se han identificado abundantes estudios realizados en otras regiones del mundo, como América del Sur^40^^,^^41^^,^^43^ o Asia^46^.

Todos los estudios utilizaron al menos tres instrumentos para valorar la efectividad de las intervenciones realizadas. Analía Verónica Losada y Julieta Marmo indican la necesidad de aplicar diversos instrumentos de evaluación en los casos de TCA a fin de conocer distintos aspectos habitualmente implicados, no solo para definir un diagnóstico puntual sino también para adaptar el tratamiento de acuerdo a las áreas que puedan verse afectadas^47^.

Coincidiendo con la mayoría de estudios de la revisión, las intervenciones publicadas suelen ser colectivas y dirigidas a poblaciones específicas: escolares^43^, personas universitarias^42^^,^^45^, pacientes hospitalizadas diagnosticadas con TCA^44^ o usuarias de gimnasios^40^.

Tres estudios incluyeron el uso de fotografías en sus intervenciones^34^^,^^35^^,^^39^, como también lo hicieron Ojeda-Martín y col^3^ para valorar si la exposición a los contenidos y fotografías de las RRSS influían negativamente en el riesgo de desarrollar TCA en jóvenes, o Jorge Emiro Restrepo y Tatiana Castañeda Quirama^40^, quienes incorporaron aspectos de fotografía como los *selfies* y el uso de *Photoshop* en siete de las ocho preguntas de su encuesta sobre uso de RRSS.

La necesidad de implementar formación y educación para la salud como medida de prevención y de protección frente a los TCA^48^^,^^49^ fue tenida en cuenta por seis estudios^32^^-^^35^^,^^37^^,^^39^. Estas medidas son fundamentales no solo para estar correctamente informados sobre la alimentación y la salud, sino para mejorar las capacidades de análisis y crítica respecto a la información divulgada por diversos medios^48^.

Autores como Ruiz^49^ han planteado la necesidad de promover programas de prevención que incluyan intervenciones que trabajen el autoconcepto, la autopercepción y la autoestima, como aplicaron cinco de los estudios de la revisión^32^^,^^33^^,^^36^^-^^38^.

Noelia Lozano-Muñoz y col^1^ describen que las RRSS permiten crear círculos de iguales que pueden alentar y favorecer conductas que incentiven la delgadez extrema y, por tanto, aumentar el riesgo de TCA, lo que sustentaría la relación observada por cinco estudios entre la exposición a ideales de belleza irreales en las RRSS y la preocupación e insatisfacción por la imagen corporal^33^^-^^36^^,^^38^.

Las mujeres presentaron mayor auto objetivación e internalización del ideal de belleza delgado^32^^,^^33^, internalización que no solo predice cambios a nivel cognitivo sino que permite medir otros factores de riesgo como pueden ser el inicio de comportamientos compensatorios^50^. Las RRSS parecen afectar la interiorización del ideal de belleza delgado^33^, por lo que es necesario conocer las consecuencias de su uso frecuente en pacientes con TCA (la exposición a imágenes de alta carga comparativa en las RRSS disminuye la autoestima y aumenta la ansiedad), pero también promover un uso sano y adecuado^51^ mediante alfabetización sobre las RRSS, como la implementada por Sian A McLean y col^38^.

Solo un estudio abordó la imagen corporal, el manejo nutricional y la autoestima^36^, tres aspectos que distintos autores recomiendan incluir para mejorar la satisfacción corporal^52^, mejorar los parámetros de restricción dietética^53^ e incorporar factores protectores frente a la insatisfacción corporal u otros trastornos psicológicos^54^.

La presente revisión sistemática muestra algunas limitaciones, como haber limitado la estrategia de búsqueda a los últimos diez años. Esto ha imposibilitado recuperar toda la información sobre el tema de la investigación, aunque el riguroso proceso seguido ha permitido acceder a la evidencia científica más actualizada en un campo, el de las RRSS, que está en continua evolución y cuyas fechas de lanzamiento se remontan a menos de 20 años: 2004 para Facebook^55^, 2006 para *Twitter* (ahora X) ^56^, 2010 para Instagram^57^, 2011 para Snapchat^58^ y 2017 para Tik-Tok^59^ (RRSS incluidas en esta revisión). Otra limitación es que algunos estudios no centraron sus investigaciones en una RRSS en particular, realizando una valoración general del impacto. A pesar de ello, el resto de los estudios incluidos sí han identificado las RRSS que han formado parte de sus investigaciones. Todo ello nos ha permitido analizar el impacto de las intervenciones para mitigar la influencia de las RRSS en la AN y BN.

En conclusión, la mayoría de los estudios se centraron en población adolescente femenina y evidenciaron la necesidad de formar a la población para aumentar el nivel de conocimientos y reducir el riesgo de TCA. Para ello, es necesario aplicar intervenciones de carácter educativo centradas en prevención y promoción de la salud, autocrítica, autopercepción, autoestima, imagen corporal, manejo nutricional y alfabetización digital, esta última a fin de usar mejor las RRSS y reducir su impacto negativo respecto de los TCA. Se ha observado una correlación positiva entre el nivel de exposición a ideales de belleza irreales en las RRSS y mayor preocupación e insatisfacción por la imagen corporal, especialmente en mujeres, ya que presentan mayores niveles de internalización del ideal de belleza delgado y de auto objetivación. Dada la edad de los participantes, las intervenciones se aplicaron mayoritariamente en centros de educación secundaria y centros universitarios. Tras ellas, los participantes expresaron sentirse menos influidos por los medios y las RRSS, al mismo tiempo que mejoraron su autopercepción y autoestima -especialmente en mujeres-, lo que redujo los niveles de ansiedad. También disminuyeron la interiorización del ideal de belleza delgado, las restricciones dietéticas y la comparación entre usuarios de RRSS.

En un futuro las investigaciones deberían profundizar en las técnicas de alfabetización digital para promover un mejor uso de las RRSS y analizar el impacto de los *influencers* en la difusión de contenido sobre salud, alimentación y cuidado personal.
